# Buckwheat Honey Attenuates Carbon Tetrachloride-Induced Liver and DNA Damage in Mice

**DOI:** 10.1155/2015/987385

**Published:** 2015-10-05

**Authors:** Ni Cheng, Liming Wu, Jianbin Zheng, Wei Cao

**Affiliations:** ^1^Department of Food Science and Engineering, School of Chemical Engineering, Northwest University, 229 North TaiBai Road, Xi'an 710069, China; ^2^Shaanxi Provincial Key Lab of Electroanalytical Chemistry, Institute of Analytical Science, Northwest University, 229 North TaiBai Road, Xi'an 710069, China; ^3^Institute of Apicultural Research, Chinese Academy of Agricultural Science, Beijing 100093, China

## Abstract

Buckwheat honey, which is widely consumed in China, has a characteristic dark color. The objective of this study was to investigate the protective effects of buckwheat honey on liver and DNA damage induced by carbon tetrachloride in mice. The results revealed that buckwheat honey had high total phenolic content, and rutin, hesperetin, and *p*-coumaric acid were the main phenolic compounds present. Buckwheat honey possesses super DPPH radical scavenging activity and strong ferric reducing antioxidant power. Administration of buckwheat honey for 10 weeks significantly inhibited serum lipoprotein oxidation and increased serum oxygen radical absorbance capacity. Moreover, buckwheat honey significantly inhibited aspartate aminotransferase and alanine aminotransferase activities, which are enhanced by carbon tetrachloride. Hepatic malondialdehyde decreased and hepatic antioxidant enzymes (superoxide dismutase and glutathione peroxidase) increased in the presence of buckwheat honey. In a comet assay, lymphocyte DNA damage induced by carbon tetrachloride was significantly inhibited by buckwheat honey. Therefore, buckwheat honey has a hepatoprotective effect and inhibits DNA damage, activities that are primarily attributable to its high antioxidant capacity.

## 1. Introduction

The liver plays important roles in metabolism, secretion, excretion, and biotransformation. In China, where liver disease is common, there are approximately 130 million individuals with hepatitis B virus, which may contribute to chronic hepatitis, cirrhosis, or liver cancer. Therefore, there has been an increasing interest in the treatment and prevention of liver disease. Oxidative stress, which is involved in the pathogenesis of liver diseases, leads to hepatic damage [1]. Antioxidants such as silymarin, tocopherol, and betaine have desirable effects in patients with liver disease [2–4].

Carbon tetrachloride (CCl_4_) is one of the most widely used toxins for the experimental induction of liver damage in laboratory animals. The hepatotoxicity of CCl_4_ stems from reductive dehalogenation products, such as trichloromethyl (CCl_3_^∙^) and trichloromethyl peroxyl (CCl_3_O_2_^∙^) radicals [5], which can bind to proteins and lipids or remove a hydrogen atom from an unsaturated lipid, thereby initiating lipid peroxidation and contributing to liver damage [6]. In recent years, numerous studies have shown that polyphenol extract from natural products with high scavenging radical activity and strong reducing power could attenuate CCl_4_-induced liver damage [7–9]. Our previous studies have also proven that bee pollen extract rich in phenolic compounds increases antioxidant potential in mice and protects against CCl_4_-induced liver damage [10].

Buckwheat (*Fagopyrum esculentum* Moench), which is cultivated in several Asian and European counties, is an important source of nectar and pollen for bees. Buckwheat honey has a characteristic dark color and its antioxidant activity has been studied for more than 10 years. Pasini et al. [11] reported that there are 20 phenolic acids in buckwheat honey, including* p*-hydroxybenzoic and* p*-coumaric acids. Phenolic antioxidants from buckwheat honey are bioavailable and increase the antioxidant activity of plasma. Gheldof et al. [12] reported that the serum antioxidant capacity determined by oxygen radical absorbance capacity (ORAC) was significantly increased following the consumption of buckwheat honey in water. However,* in vitro* serum lipoprotein oxidation and thiobarbituric acid reactive substances (TBARS) were not significantly affected following a single consumption of buckwheat honey. Therefore, long-term studies on oxidative stress-induced illnesses are necessary to investigate whether buckwheat honey has antioxidant-related health benefits. In this study, we assessed the antioxidant capacity of buckwheat honey in mice and evaluated its protection potential for attenuating CCl_4_-induced liver and DNA damage.

## 2. Materials and Methods

### 2.1. Materials

Buckwheat honey was obtained from Shaanxi Bee Master Co., Ltd. (Xi'an, China). The pollen frequency (*Fagopyrum esculentum*) was approximately 61%. Buckwheat honey samples were stored at 4°C.

### 2.2. Chemicals and Reagents

Fluorescein disodium (FL), 1,1-diphenyl-2-picrylhydrazyl radical 2,2-diphenyl-1-(2,4,6-trinitrophenyl)hydrazyl (DPPH), 2,2′-azobis(2-amidino-propane)dihydrochloride (AAPH), dimethyl sulfoxide (DMSO), Trolox, and silymarin were obtained from Sigma-Aldrich (Steinheim, Germany). Agarose was purchased from BioRad (Hercules, CA, USA). Diagnostic kits for aspartate aminotransferase (AST), alanine aminotransferase (ALT), malondialdehyde (MDA), superoxide dismutase (SOD), glutathione peroxidase (GSH-Px), and protein were obtained from Nanjing Jiancheng Bioengineering Institute (Nanjing, China). Lymphocyte separation medium was purchased from Tianjin Hao Yang Biological Manufacture Co., Ltd. CCl_4_, peanut oil, and other chemicals were acquired from Tianjin Kemiou Chemical Reagent Co. (Tianjin, China).

### 2.3. Antioxidant Assays

#### 2.3.1. Total Phenolic Content (TPC) and HPLC Analysis

We used a modified Folin-Ciocalteu method to determine TPC in buckwheat honey [13]. Briefly, 0.2 mg of buckwheat honey was mixed with 1.0 mL of Folin-Ciocalteu reagent, allowed to stand at room temperature for 5 min, and mixed with 5 mL of 1 M Na_2_CO_3_. An hour later, absorbance was measured at 760 nm. TPC was expressed as the gallic acid equivalents per gram of buckwheat honey (mg GA/g).

The contents of individual phenols in buckwheat honey were estimated by HPLC-DAD analysis as proposed by Liang et al. [14]. An Agilent 1100 HPLC system (Agilent Technologies Deutschland, Waldbronn) equipped with a vacuum degasser, a quaternary solvent delivery pump, a manual chromatographic valve, a thermostated column compartment, and a diode-array detector (Agilent, Palo Alto, CA, USA) was used. The column was a Zorbax SB-C18 column (150 mm × 4.6 mm, 5.0 *μ*m). The mobile phase adopted was methanol (A) and 0.15% aqueous acetic acid solution (B) (v/v) using a linear gradient elution of 5–15% A at 0–10 min, 15–35% A at 10–15 min, 35–55% A at 15–20 min, 55–65% A at 20–25 min, 65–80% A at 25–30 min, and 80% A at 30–35 min. The injected volume was 5 *μ*L, and flow rate was 1.0 mL/min. The column was operated at 30°C. The diode-array detector was performed at 360 nm.

#### 2.3.2. DPPH Radical Scavenging Activity

DPPH radical scavenging activity of buckwheat honey was assessed according to the method proposed by Wang et al. [15]. Briefly, different volumes of buckwheat honey (0.2 g/mL) were mixed with 4.0 mL of 0.1 mM DPPH radical solution. After adjusting the total volume to 10 mL, the mixture was mixed well and allowed to stand at room temperature for 30 min in the dark. Absorbance was measured at 517 nm. The DPPH radical scavenging activity was expressed as Trolox equivalents per gram of buckwheat honey (mg Trolox/g).

#### 2.3.3. Ferrous Ion-Chelating Activity

The ferrous ion-chelating activity of buckwheat honey was measured by the method reported by Singh and Rajini with some modifications [16]. In this experiment, 50 *μ*L of buckwheat honey (0.2 g/mL) was mixed with 50 *μ*L of 1 mM iron vitriol and 20 *μ*L of 1 mM ferrozine. The total volume was adjusted to 1 mL with methanol and incubated at room temperature for 10 min. The absorbance of the ferrozine-Fe^2+^ complex was measured at 562 nm. Ferrous ion-chelating activity was expressed as Na_2_EDTA equivalents per gram of buckwheat honey (mg Na_2_EDTA/g).

#### 2.3.4. Ferric Reducing Antioxidant Power (FRAP)

FRAP of buckwheat honey was assessed by the method reported by Benzie and Strain [17]. Buckwheat honey (0.3 mL at 0.2 mg/mL) was mixed with 4.0 mL of FRAP reagent (2.5 mL of 10 mM TPTZ solution in 40 mM HCl with 2.5 mL of 20 mM FeCl_3_; 25 mL of 0.3 M acetate buffer, pH 3.6), mixed well and incubated at 37°C for 4 min. Absorbance was measured at 593 nm. FRAP was expressed as Trolox equivalents per gram of buckwheat honey (mg Trolox/g).

#### 2.3.5. Animals and Study Design

*(1) Animals*. Male Kunming mice (18–22 g) were obtained from Xi'an Jiaotong University and housed in cages with six mice per cage. The animal ethical approval communication number is SCXK 2012-003. The animal experiments followed the guidelines and regulations of the State Committee of Science and Technology of the People's Republic of China.

After acclimatization to laboratory conditions for 7 d, the mice were randomly divided into four groups (12 mice/group). Control mice and CCl_4_-treated mice were administered distilled water via gavage at 0.22 mL/10 g BW, twice daily for 10 weeks. According to the doses of honey and silymarin reported by Cheng et al. [18], the mice were administered 0.22 g/10 g BW of buckwheat honey and 0.5 mg/10 g BW of silymarin via gavage twice daily for 10 weeks. To investigate the serum antioxidant capacity after administration of buckwheat honey, the mice in the control and honey groups were bled by cardiac puncture 2 h after the last administration. The blood samples were centrifuged at 3000 rpm for 15 min to obtain serum. The serum was used for serum lipoprotein oxidation and ORAC assays.

To investigate the protective effects of buckwheat honey on CCl_4_-induced liver damage, all mice were continuously intragastrically administered distilled water, buckwheat honey, and silymarin for the next week. Two hours following the last administration, all mice (except control mice) were administered a CCl_4_/peanut oil mixture (0.2 : 100, intraperitoneally, 0.1 mL/10 g BW); control mice received only peanut oil. Subsequently, the animals were fasted for 16 h and bled by cardiac puncture. Half of the blood samples were transferred to anticoagulant tubes for separating lymphocytes, and the other half were transferred to ordinary centrifuge tubes for serum collection.

*(2) Serum Lipoprotein Oxidation*. Serum lipoprotein oxidation was assessed by the method reported by Regnström et al. [19]. Serum samples from control and honey groups were diluted with phosphate buffer (10.1 mM Na_2_HPO_4_, 1.8 Mm KH_2_PO_4_, 27 mM KCl, and 138 mM NaCl) to 0.5%. Copper ions at 12 *μ*mol/L were added to the diluted serum samples. Oxidation kinetics was determined at 234 nm every 20 minutes at 37°C. Diluted serum samples without copper were used as blanks. The area under the oxidation curve (AUC) was plotted and the percentage inhibition of serum lipoprotein oxidation was calculated according to the following equation:

(1) $$\begin{array}{c} Inhibition\left(\;\%\;\right)=\frac{\left({AUC}_{control}-{AUC}_{honey}\right)}{{AUC}_{control}}\times 100, \end{array}$$

where AUC_control_ is the area under the oxidation curve for the control group serum samples and AUC_honey_ is the area under the oxidation curve for the honey group serum samples.

*(3) ORAC Assay*. The ORAC assay was performed in 96-well plates and measured in a multifunctional plate reader (Infinite M200Pro, Switzerland) [20]. Serum samples from the control and honey groups were used in this assay. Analyses were performed in 75 mM sodium phosphate buffer (pH = 7.4) at 37°C. The excitation wavelength was 485 nm and the emission wavelength was 535 nm. FL was used as the substrate and AAPH was used for the production of peroxyl radicals. Briefly, 50 *μ*L of 78 nM FL and 50 *μ*L of 1% serum were transferred to 96-well plates. The blank consisted of 50 *μ*L of phosphate buffer instead of serum. The mixture was preincubated at 37°C for 30 min before rapidly adding 25 *μ*L of 221 mM AAPH solution. The plate was automatically shaken prior to each reading. Fluorescence was measured every 5 minutes. The assay was performed in triplicate, and the results were expressed as inhibition of the area under the curve (AUC) according to the following equation:

(2) Inhibition%=net  AUChoney−net  AUCcontrolnet  AUCcontrol×100,

where net AUC_control_ = AUC_control_ − AUC_blank_; net AUC_honey_ = AUC_honey_ − AUC_blank_.

*(4) Comet Assay*. The comet assay is the preferred technique for detecting DNA damage in single cells. In this study, lymphocytes isolated from control mice, CCl_4_-treated mice, and honey mice were analyzed by the comet assay to assess the protective effects of buckwheat honey on CCl_4_-induced DNA damage. Lymphocytes from silymarin mice were set as the positive reference. Following the methods proposed by Singh et al. [21] with slight modifications, lymphocytes were suspended in 0.15 M of phosphate buffer (pH 7.4) at a density of 1 × 10^5^/mL. After fixing the lymphocytes on slides, the slides were immersed in lysis buffer (2.5 M NaCl, 100 mM EDTA, 1% N-lauroylsarcosine at pH 10, 10 mM Tris-HCl, 1% Triton X-100, and 10% dimethyl sulfoxide (DMSO)) for 2 h. Subsequently, the slides were immersed in electrophoresis buffer (1 mM EDTA and 300 mM NaOH, pH 13) for DNA unwinding. After 30 min, electrophoresis was run at 25 V (300 mA) for 20 min in the dark. All slides were treated with ethidium bromide and observed under a fluorescence microscope (Nikon 027012; Nikon, Tokyo, Japan). The results were scored and analyzed using an automated analysis system of the Comet Assay Software Project (CASP). At least 50 cells were scored from each slide. The degree of DNA damage was scored by determining the percentage of DNA in the tail (tail DNA %) and olive tail moment (OTM), defined as the fraction of tail DNA multiplied by the distance between the means of the head and tail:

(3) tail  DNA%=tail  DNAhead  DNA+tail  DNA×100,OTM=tail  DNA%×tail  mean−head  mean.

*(5) Assessment of Liver Function*. Serum was obtained following the centrifugation of blood samples at room temperature for 20 min at 3,000 rpm. Serum ALT and AST values were measured using commercially available diagnostic kits.

*(6) Determination of MDA, SOD, and GSH-Px Activities*. After the animals were sacrificed, livers were immediately excised. With the exception of a portion of the left lobe to be used for histopathological examination, the livers were homogenized in phosphate buffer (50 mM, pH 7.4) and centrifuged at 2,500 rpm for 20 min at 4°C. The MDA content, SOD, and GSH-Px activities along with protein levels in the supernatant were estimated according to commercially available diagnostic kits.

*(7) Histopathological Examinations*. A left lobe portion of the liver was incubated for 24 h in 10% neutral formalin solution. Based on standard procedures, we obtained 5 *μ*m sections for histopathological studies using hematoxylin and eosin (H&E) staining.

### 2.4. Statistical Analysis

We analyzed the data in triplicate using SAS software version 8.1 (SAS Institute, Cary, NC, USA). Tukey's posttest was used to assess statistical significance (*P* value < 0.05).

## 3. Results

### 3.1. Antioxidant Assay of Buckwheat Honey

**Figure 1:**
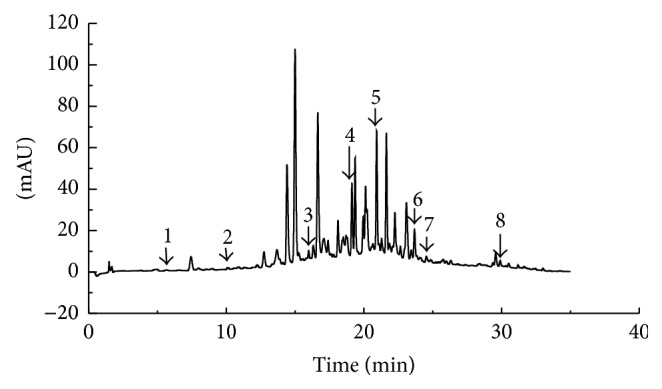
Chromatogram of the buckwheat honey using HPLC-DAD. Peaks: 1 = gallic acid; 2 = protocatechuic acid; 3 = chlorogenic acid; 4 =* p*-coumaric acid; 5 = rutin; 6 = quercetin; 7 = hesperetin; 8 = galangin.

To study the antioxidant activity, the TPC and individual phenolic compounds of buckwheat honey were determined, and the results are shown in Table 1 and Figure 1. The TPC of buckwheat honey was 2.04 mg GA/g. Four phenolic acids and four flavones were identified in buckwheat honey. Rutin, the most abundant phenolic compound, was measured at 35.94 mg/kg, followed by hesperetin (23.76 mg/kg) and* p*-coumaric acid (12.52 mg/kg).

The results of antioxidant activities of buckwheat honey* in vitro* are shown in Table 2. The DPPH radical scavenging activity is a widely used method to evaluate antioxidant capacity. The DPPH radical scavenging activity of buckwheat honey was 0.304 mg Trolox/g. The ferrous ion-chelating activity of buckwheat honey was 0.479 mg Na_2_EDTA/g. The FRAP assay is often used to determine the antioxidant properties of foods based on their electron-donating capacity [22]. As shown in Table 2, the FRAP value of buckwheat honey was 0.355 mg Trolox/g, which is comparable to the values obtained in jujube honey, but lower than those obtained in cacao farm honey, mangrove honey, citrus honey, and a coconut grove honey in Mexico (48–152 mg Trolox/100 g) [23].

### 3.2. Buckwheat Honey Increased Serum Antioxidant Capacity in Mice

**Figure 2:**
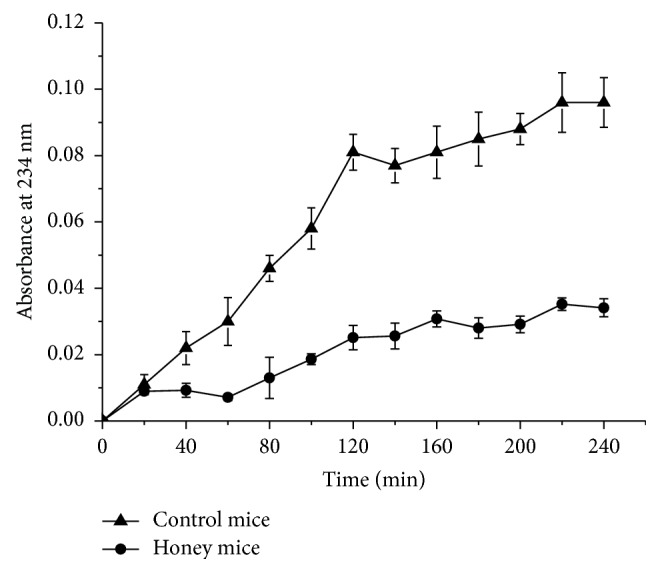
Effects of buckwheat honey on serum lipoprotein oxidation (absorbance values had been adjusted for the initial absorbance reading). Control mice were administered distilled water via gavage. Honey mice were administered buckwheat honey (0.22 g/10 g BW, twice daily for 10 weeks) via gavage.

**Figure 3:**
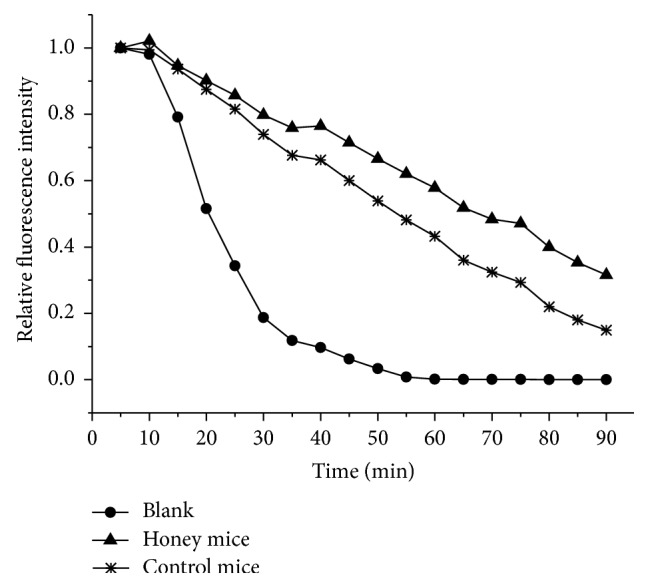
Effects of buckwheat honey on serum oxygen radical absorbance capacity (ORAC). Control mice were administered distilled water via gavage. Honey mice were administered buckwheat honey (0.22 g/10 g BW, twice daily for 10 weeks) via gavage. In the blank, PBS was used instead of serum.

The administration of buckwheat honey (0.22 g/10 g BW, twice daily) for 10 weeks resulted in the inhibition of serum lipoprotein oxidation. Buckwheat honey inhibited serum lipoprotein oxidation by 65.71% (Figure 2). Serum ORAC is another method for measuring serum antioxidant capacity. As described in Figure 3, serum from honey-treated mice had a relatively high ORAC value, whereas serum from control mice had a relatively low ORAC value (27.19% lower than the former).

### 3.3. Buckwheat Honey Attenuated DNA Damage Induced by Carbon Tetrachloride

**Figure 4:**
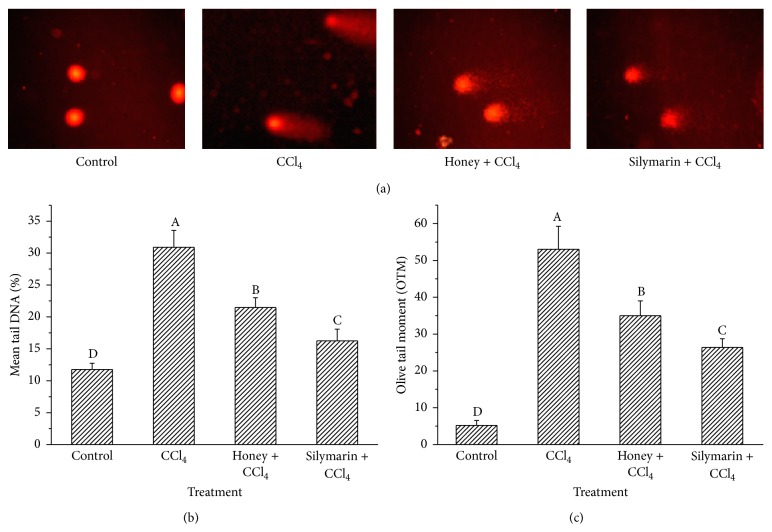
Effects of buckwheat honey on mice lymphocyte DNA damage induced by CCl_4_ ((a) picture of lymphocyte DNA damage; (b) mean tail DNA%; (c): olive tail moment). Control: lymphocytes from control mice; CCl_4_: lymphocytes from CCl_4_-treated mice; honey + CCl_4_: lymphocytes from mice administered buckwheat honey (0.22 g/10 g BW) twice daily for 11 weeks prior to CCl_4_; silymarin + CCl_4_: lymphocytes from mice administered silymarin (0.5 mg/10 g BW) twice daily for 11 weeks prior to CCl_4_.

The protective effect of buckwheat honey on CCl_4_-induced damage is shown in Figure 4. Based on the picture of lymphocytes in the CCl_4_-treated group, a significant increase in the tail length of comet was observed. However, the lymphocyte from the mice administered honey and silymarin showed a similar decrease in the tail length of comet. As shown in Figures 4(b) and 4(c), a similar variation was presented in mean tail DNA and OTM. The mean tail DNA and OTM in the CCl_4_-treated group were 30.91% and 53.03%, respectively, whereas the mean tail DNA and OTM in the control group were 11.76% and 5.21%, respectively. Therefore, significant increases in the mean tail DNA and OTM of lymphocytes were associated with CCl_4_ exposure (*P* < 0.05). Interestingly, pretreatment with buckwheat honey (0.22 g/10 g BW, twice daily) for 11 weeks decreased lymphocyte damage significantly (*P* < 0.05). Silymarin, as a positive reference, had super protective effect on DNA damage induced by CCl_4_.

### 3.4. Buckwheat Honey Protected the Liver from Carbon Tetrachloride-Induced Damage

**Figure 5:**
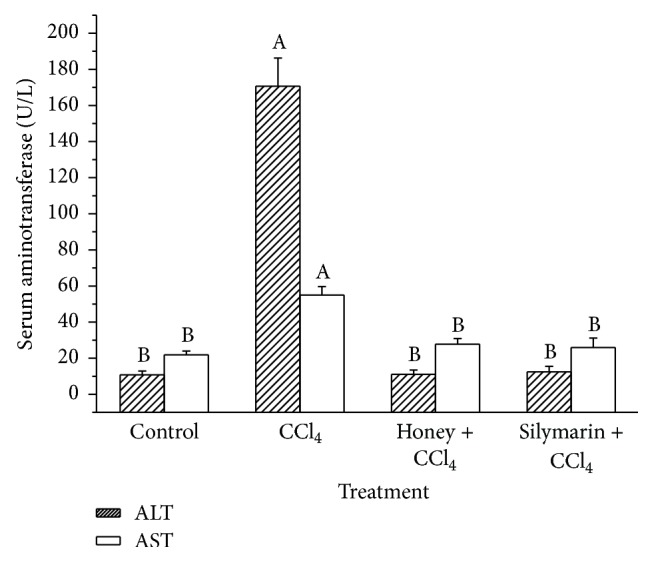
Effects of buckwheat honey on serum ALT and AST activities. Different lower case letters represent significant differences (*P* < 0.05). Mice in “control”: distilled water plus peanut oil; mice in “CCl_4_”: distilled water plus CCl_4_; mice in “honey + CCl_4_”: buckwheat honey (0.22 g/10 g BW) twice daily for 11 weeks plus CCl_4_; mice in “silymarin + CCl_4_”: silymarin (0.5 mg/10 g BW) twice daily for 11 weeks plus CCl_4_.

Serum ALT and AST activities were determined in this study and the results are shown in Figure 5. In the CCl_4_-treated group, serum ALT and AST activities were 170.68 and 55.01 U/L, which were 15x and 1.52x higher than those of the control group, respectively (*P* < 0.05). In the honey and silymarin groups, serum ALT and AST activities were 11.12 and 27.77 U/L and 12.43 and 25.96 U/L, respectively. There were no significant differences in the hepatic enzyme activities between the control, honey, and silymarin groups. Therefore, buckwheat honey treatment (0.22 g/10 g BW, twice daily) for 11 weeks inhibited an increase in serum ALT and AST activity.

**Figure 6:**
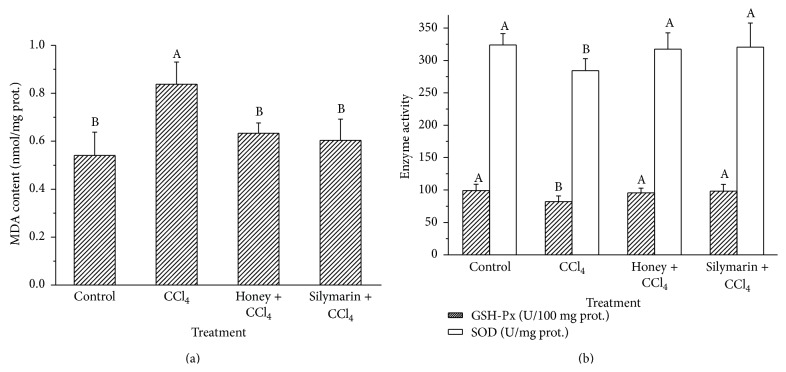
Effects of buckwheat honey on hepatic MDA content (a) and GSH-Px and SOD activities (b). Different lower case letters represent significant differences (*P* < 0.05). Mice in “control”: distilled water plus peanut oil; mice in “CCl_4_”: distilled water plus CCl_4_; mice in “honey + CCl_4_”: buckwheat honey (0.22 g/10 g BW) twice daily for 11 weeks plus CCl_4_; mice in “silymarin + CCl_4_”: silymarin (0.5 mg/10 g BW) twice daily for 11 weeks plus CCl_4_.

Hepatic MDA levels and GSH-Px and SOD activities were monitored in this study and the results are shown in Figure 6. A 54.90% increase of hepatic MDA was obtained in the CCl_4_-treated group relative to the control mice. Pretreatment with buckwheat honey (0.22 g/10 g BW, twice daily) and silymarin (0.5 mg/10 g BW, twice daily) for 11 weeks significantly decreased hepatic MDA levels in the CCl_4_-treated mice (*P* < 0.05) (Figure 6(a)). The activities of GSH-Px and SOD in CCl_4_-treated mice decreased significantly compared to the control mice (*P* < 0.05; Figure 6(b)). Interestingly, pretreatment with buckwheat honey and silymarin significantly inhibited the decrease in GSH-Px and SOD activities induced by CCl_4_ (*P* < 0.05).

**Figure 7:**
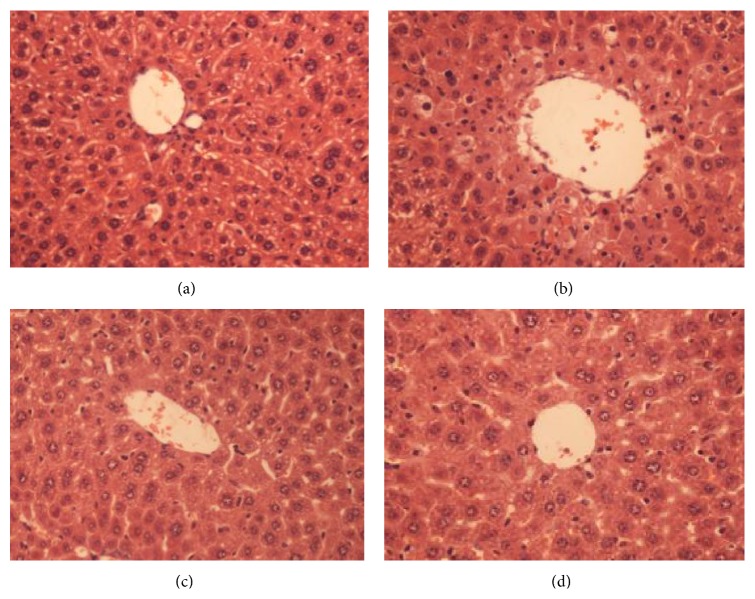
Effects of buckwheat honey on hepatic morphological analysis (×400 H&E): control mice (a), CCl_4_-treated mice (b), and mice pretreated with buckwheat honey prior to CCl_4_ (c) and with silymarin (d).

The histological observations supported the results obtained from the enzyme assays. Liver sections from control mice showed regular cellular morphology (Figure 7(a)). However, liver sections from CCl_4_-treated mice revealed extensive liver damage characterized by severe hepatocellular hydropic degeneration and necrosis around the central vein, dilated sinusoidal spaces, inflammatory cell infiltration, and ballooning degeneration (Figure 7(b)). Surprisingly, pretreatment with buckwheat honey remarkably ameliorated the hypertrophy of hepatocytes, inflammatory cell infiltration, ballooning degeneration, and dilated sinusoidal spaces (Figure 7(c)). The protective effect of buckwheat honey was similar to silymarin (Figure 7(d)).

## 4. Discussion

Phenolic compounds are present in plants and food products, including honey. Phenolic compounds possess powerful antioxidant capacity by acting as hydrogen donors to free radicals and as electron donors to metal ions [22]. According to previous studies, phenolic compounds are the main contributor to the antioxidant activity of honey. Moreover, the darker the honey, the stronger its antioxidant capability. Buckwheat honey is deemed the darkest honey in China. Therefore, a higher value of TPC (2.04 mg GA/g) was acquired in this study, which was significantly higher than that of seven honey samples from Slovenia, which ranged from 44.8 mg GA/kg in acacia honey to 241.4 mg GA/kg in fir honey [24]. Because phenolic hydroxyl can donate a hydrogen atom to reduce free radicals [25], the DPPH radical scavenging activity of buckwheat honey was higher than that reported in black locust honey (0.3 mmol Trolox/kg), goldenrod honey (0.2 mmol Trolox/kg), rapeseed honey (0.4 mmol Trolox/kg), and heather honey (0.6 mmol Trolox/kg) [26]. The ferrous ion-chelating activity represents another index of antioxidant activity in bioactive compounds because divalent transition metal ions play important roles in oxidation, such as by contributing to the formation of hydroxyl radicals and hydroperoxides via the Fenton reaction [27]. The ferrous ion-chelating activity of buckwheat honey was 0.479 mg Na_2_EDTA/g, approximately 10x higher than that of jujube honey (37.59–53.04 mg Na_2_EDTA/kg) [18]. The metal-chelating potential is strongly dependent on the arrangement of hydroxyls and carbonyl groups around the molecule [25]. Flavonoids such as rutin and hesperetin have been identified in buckwheat honey as the main phenolic compounds. Therefore, it is not difficult to understand why buckwheat honey has a high ferrous ion-chelating activity.

To investigate whether buckwheat honey could increase the antioxidant capacity of the mice, Cu^2+^-induced oxidation of serum lipoprotein was determined. This method provides an indication of diene formation in lipoprotein fatty acids when exposed to Cu^2+^. Diene formation is assessed by measuring changes in absorbance at 234 nm. High absorbance values correspond to diene formation as a result of serum lipoprotein oxidation, and low absorbance values correspond to inhibition of serum lipoprotein oxidation and, consequently, to high antioxidant activity [28]. The administration of buckwheat honey remarkably inhibited serum lipoprotein oxidation in this study. Phenolic compounds present in buckwheat honey inhibit oxidation of serum lipoproteins by acting as free radical scavengers or as metal-chelating agents [20]. Antioxidants are often added to foods to prevent the radical chain reactions of oxidation, and they act by inhibiting the initiation and propagation step, leading to termination of the reaction and a delay in the oxidation process [25, 29]. Therefore, buckwheat honey significantly inhibited the Cu^2+^-induced oxidation of serum lipoprotein and increased the antioxidant capacity of mice. Serum ORAC is another method for measuring serum antioxidant capacity. This method, which incorporates FL as the fluorescent probe, is commonly used in biological samples and foods. The ORAC method is based on the inhibition of peroxyl-radical-induced oxidation initiated by the thermal decomposition of AAPH [20]. FL blocks the peroxyl-radical chain reaction process by donating hydrogen protons, thereby reducing the fluorescence intensity. Antioxidants can inhibit the decrease in fluorescence intensity by scavenging AAPH or by donating hydrogen protons, thereby blocking the free radical chain reaction [30]. Accordingly, buckwheat honey administered to mice for 10 weeks at 0.22 g/10 g BW increased the serum antioxidant activity.

Intraperitoneal administration of CCl_4_ is a classic method used to induce oxidation and liver damage [7, 8]. Metabolites of CCl_4_ include highly reactive free radicals, which initiate the chain reaction of lipid peroxidation, thereby affecting polyunsaturated fatty acids and phospholipids [31]. Lipid peroxidation affects the permeability of the mitochondria, endoplasmic reticulum, and plasma membranes, resulting in leakage of hepatic enzymes in the blood. Serum ALT and AST activities have been confirmed to be the most sensitive indicator of CCl_4_-induced liver damage. Therefore, serum ALT and AST activities in the CCl_4_-treated group were significantly higher than those of the control group. Interestingly, pretreatment with buckwheat honey inhibited the increase of serum ALT and AST activities induced by CCl_4_. With increasing serum ALT and AST activities as a result of CCl_4_-induced damage, lipid peroxidation products (e.g., MDA) accumulate in hepatic cells. Therefore, hepatic MDA levels were monitored in this study. As shown in Figure 6(a), a 54.90% increase in hepatic MDA was obtained in the CCl_4_-treated group relative to the control mice. Pretreatment with buckwheat honey and silymarin significantly decreased hepatic MDA levels in the CCl_4_-treated mice. To delineate the mechanisms underlying the protective effects of buckwheat honey, the activities of hepatic antioxidant enzymes (e.g., GSH-Px and SOD) were determined. In this study, the activities of GSH-Px and SOD in the CCl_4_-treated mice decreased significantly compared to those in the control mice (*P* < 0.05; Figure 6(b)). SOD is a critical endogenous antioxidant enzyme that prevents and neutralizes oxidative damage [32]. GSH-Px, which has both intracellular and extracellular antioxidant functions, catalyzes the reduction of hydrogen peroxide and hydroperoxides into nontoxic products [7]. When present in excess, lipid peroxides and reactive oxygen species can easily inactivate these antioxidant enzymes [33]. Therefore, the reduction in GSH-Px and SOD was attributed to an enhanced toxicity by CCl_4_. Interestingly, pretreatment with buckwheat honey and silymarin significantly inhibited the decrease in GSH-Px and SOD activities induced by CCl_4_ (*P* < 0.05). Meanwhile, histological observations further affirmed that administration with buckwheat honey significantly attenuates CCl_4_-induced liver damage.

In the present study, eight phenolic compounds were identified in buckwheat honey, of which rutin and hesperetin are the majority. Rutin has been verified to exert renal-protective effects by inhibiting ROS and antioxidant activities [34]. Hesperetin has also been confirmed to protect against oxidative stress-related hepatic dysfunction [35]. In addition to rutin and hesperetin, there are some unidentified high content antioxidants in buckwheat, which may work together in creating these antioxidative and hepatoprotective effects. Silymarin, a high antioxidative flavonoid, has been used as a drug for human liver disease induced by oxidative stress for at least two decades [36]. Usually, it is used as a positive reference in many studies [9], including in this study on oxidative stress. Caffeic acid, unidentified in this study, was found to exist in buckwheat honey [11] and have the capability of preventing nickel-induced oxidative damage in rat livers [37]. Recently, inhibition of free radical-induced damage by antioxidant supplementation has become an attractive therapeutic strategy for reducing the risk of liver disease [7]. Polyphenol extracts from natural products such as apples,* Murraya koenigii *L., yam peel, and bee pollen have been studied for their hepatoprotective effects [7, 9, 10]. The high levels of phenolic compounds in buckwheat honey reported in previous studies [13] were confirmed in this study. Additionally, buckwheat honey has free radical scavenging and ferrous ion-chelating properties. Moreover, CCl_4_-induced DNA damage can be inhibited by the administration of buckwheat honey. Therefore, buckwheat honey, which has a free radical scavenging ability, reduces lipid peroxidation and increases antioxidant capacity, thereby attenuating CCl_4_-induced liver damage in mice.

Additionally, another aim of this study was to assess whether buckwheat honey can attenuate CCl_4_-induced DNA damage. Lymphocyte DNA damage induced by carbon tetrachloride was assessed by alkaline single cell gel electrophoresis, that is, the comet assay. This method is a rapid and sensitive technique for measuring and analyzing DNA damage in individual cells [38]. The more severe the cell damage, the higher the amount of tail DNA. Another parameter for DNA damage analysis is OTM, which is generally considered the main index of DNA damage because it provides information about the total DNA content in the tail as well as DNA migration from the comet-head. Thus, in the present experiment, OTM is taken into consideration for the interpretation of the data [39]. In the present study, increases in tail DNA and OTM induced by CCl_4_ are shown in Figures 4(b) and 4(c). This demonstrated that the intraperitoneal administration of CCl_4_ caused a significant rise in DNA damage in peripheral lymphocytes. This result could be attributed to the* in vivo* action of carbon tetrachloride metabolites, that is, trichloromethyl and/or trichloromethyl peroxy radicals, which pass from the liver to the circulatory system, or to the appearance of stimulated nuclear cells in circulation. According to Kujawska et al., oxidative damage to DNA increases by 33% in mice following intraperitoneal administration of CCl_4_ [40]. On the other hand, Kadiiska et al. reported that CCl_4_ did not increase DNA damage in rat blood leukocytes [41], which may be attributed to the dose and time of CCl_4_ poisoning. In this study, a marked rise in tail DNA and OTM was obtained in mice treated with CCl_4_. More importantly, pretreatment with antioxidants can inhibit the increase of mean tail DNA and OTM. This protection should be attributed to the phenolic compound existing in buckwheat honey, which could scavenge the free radicals produced in the metabolism of CCl_4_ and thus attenuate the damage.

In conclusion, the results of this study demonstrated that buckwheat honey increased the antioxidant capacity and attenuated CCl_4_-induced liver and DNA damage in mice. Buckwheat honey demonstrated high TPC, free radical scavenging capability, and ferric-reducing antioxidant properties. Pretreatment with buckwheat honey for 10 weeks in mice significantly increased serum antioxidant activities. Therefore, buckwheat honey exhibited hepatoprotective effects in mice. Additionally, pretreatment with buckwheat honey protected DNA from CCl_4_-induced oxidation.

## Figures and Tables

**Table 1:** Phenolic compounds (mg/kg) and TPC (mg GA/g) of buckwheat honeys.

Phenolic compounds	Concentration
Gallic acid	2.02 ± 0.52
Protocatechuic acid	1.09 ± 0.34
Chlorogenic acid	0.56 ± 0.07
*p*-Coumaric acid	12.52 ± 1.92
Rutin	35.94 ± 3.76
Quercetin	1.97 ± 0.09
Hesperetin	23.76 ± 0.31
Galangin	2.38 ± 0.18
TPC	2.039 ± 0.03

Results presented in the table are expressed as means ± standard deviation (SD) for 3 replications.

**Table 2:** Antioxidant activities of buckwheat honey *in vitro*.

Antioxidant index	Results
DPPH radical scavenging activity	0.304 ± 0.02 (mg Trolox/g)
Ferrous ion-chelating activity	0.479 ± 0.01 (mg Na_2_EDTA/g)
Ferric reducing antioxidant power	0.355 ± 0.05 (mg Trolox/g)

The results presented in the table were expressed as the mean values ± standard deviation (SD) for 3 replications.
